# High efficacy of azacitidine combined with homoharringtonine, idarubicin, and cytarabine in newly diagnosed patients with AML: A single arm, phase 2 trial

**DOI:** 10.3389/fonc.2022.1069246

**Published:** 2022-12-08

**Authors:** Jun Li, Yanqing Huang, Yue Hou, Yan Gu, Chunhua Song, Zheng Ge

**Affiliations:** ^1^ Department of Hematology, Zhongda Hospital, School of Medicine, Southeast University, Institute of Hematology Southeast University, Nanjing, China; ^2^ Hershey Medical Center, Pennsylvania State University Medical College, Hershey, PA, United States; ^3^ Division of Hematology, The Ohio State University Wexner Medical Center, the James Cancer Hospital, Columbus, OH, United States

**Keywords:** azacitidine, homoharringtonine, phase 2 trial, newly diagnosed, AML

## Abstract

**Introduction:**

This study aims to evaluate the efficacy and safety of the novel combination of Aza and HIA as the frontline induction therapy in newly diagnosed AML patients eligible for intensive chemotherapy (IC) (registered on ClinicalTrials.gov, number NCT04248595).

**Methods:**

Aza (75mg/m2/d on days1-5 subcutaneous) is administered in combination with HIA [HHT 2mg/m2/d on days 4-8 intravenous over 3 hours, idarubicin 6mg/m2/d on days 4-6 intravenous, and cytarabine 100mg/m2/d on days 4-10 intravenous]. The primary endpoint was complete remission (CR) or CR with incomplete blood count recovery (CRi). Secondary endpoints were overall survival (OS), relapse-free survival (RFS), and adverse events (AEs).

**Results:**

A total of 20 AML patients (aged 18-70 years) were enrolled between Jan 2020 and Sep 2022. 95% (19/20) of patients achieved CR/CRi, and 89.5% (17/19) had undetectable MRD, in which 94.7% (18/19) reached CR/CRi, and 88.9% (16/18) obtained MRD negative after the 1st cycle of induction therapy. Median OS and RFS were both not reached during the follow-up. The estimated 2-year OS and RFS were 87.5% (95%CI, 58.6% to 96.7%) and 87.1% (95%CI, 57.3% to 96.6%), respectively. No patient discontinued the treatment for AEs.

**Discussion:**

This study provides preliminary evidence for this novel combination therapy as the first-line induction therapy for young or older AML patients fit for IC.

## Introduction

Acute myeloid leukemia (AML) is a heterogenous hematologic malignancy defined by malignant clonal expansion of hematopoietic progenitor cells with differentiation arrest (1). Treatment of AML has remained the same for nearly three decades. The backbone of treatment remains a combination that contains anthracycline (idarubicin or daunorubicin) and cytarabine-based regimens with allogeneic stem cell transplantation (allo-HSCT) (2). 3 days of idarubicin/daunorubicin followed by 7 days of cytarabine (hereafter “3 + 7”) is the standard first-line induction therapy among young and/or older patients fit for intensive chemotherapy (IC). However, complete remission (CR) occurs in 60-80% of young and 45-60% of older patients with newly diagnosed (ND) AML, and only 25% of patients remain alive after 5 years (3). Hence, it needs highly effective, well-tolerated therapy options for adult AML. Azacitidine (Aza) is a pyrimidine nucleoside analog of cytidine. Aza could target epigenetic gene silencing by inhibiting gene expression (4). Besides, Aza could also inhibit protein synthesis by incorporating RNA (5). Mono-Aza treatment in older AML patients yielded a CR rate of 30% or less and a median survival of less than 1 year (6). In recent years Aza has become the backbone for combination regimens of existing or novel treatments (lenalidomide (7), histone deacetylase inhibitor (8), venetoclax (9), FLT3 inhibitor (10), anti-CD33 antibody (11), etc.).

Homoharringtonine (HHT) is a natural plant alkaloid isolated from Cephalotaxus and exhibits an anti-leukemic effect (12). Previous studies have revealed different working mechanisms involved in HHT, including inhibiting protein synthesis by binding with the subunit of ribosome (13) and inducing apoptosis of leukemia cells by altering apoptosis-related proteins (14). In addition, recent studies have demonstrated that HHT could also exert an anti-leukemic effect by inhibiting the NF-κB signaling pathway (15) and modulating the JAK2-STAT5 signal pathway through the alteration of protein tyrosine kinase phosphorylation (16). Omacetaxine, a semisynthetic form of HHT, was approved by US FDA for treating chronic myeloid leukemia (CML) refractory to TKIs in 2012 (17). ND CML patients or failed prior therapy (imatinib, IFN-α) could benefit from receiving HHT-based regimens (18–21). It is reported that HHT in combination with cytarabine, aclarubicin/daunorubicin (HAA/HDA) achieved a higher CR rate than the standard “3+7” regimen (DA) in treating *de novo* AML (22). Our recently published clinical trial demonstrated promising therapeutic responses in elderly AML patients or ineligible for IC treated with Aza combined with HAG (HHT, low-dose cytarabine, G-CSF) (23). However, so far, it has not been well determined the efficacy and safety of adding Aza to the HHT-based regimen for young and/or older patients eligible for IC as the first-line inducting therapy.

Here, we reported a phase 2 clinical trial for a novel combination of Aza with HIA regimen (homoharringtonine, idarubicin, cytarabine) in ND patients with AML. The results demonstrated that this combined regimen has high efficacy for young or older patients fit for IC as first-line induction therapy. These preliminary results supported a further multi-center, phase 3 clinical trial with a larger sample.

## Patients and methods

### Clinical trial design and participants

This is a single center, single arm, phase 2 trial at Zhongda Hospital (Nanjing, China). This trial was registered on ClinicalTrials.gov, number NCT04248595.

**Figure 1:**
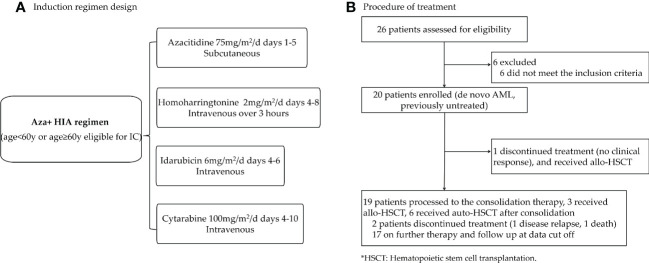
Trial profile **(A)** Clinical design of Aza+HIA induction regimen. (Aza, azacitidine; HIA, homoharringtonine, idarubicin, cytarabine). **(B)** A total of 26 AML patients were screened, 6 of them were not stratified according to the eligibility for receiving IC. Finally, 20 patients were enrolled in this trial.

A total of 20 AML patients (aged 18-70 years) diagnosed by the 2016 World Health Organization criteria (WHO) (24) were enrolled between Jan 2020 and Sep 2022. All of the enrolled patients were ND, untreated *de novo* AML (no history of the myelodysplastic syndrome (MDS), myeloproliferative neoplasms (MPN), chronic myelomonocytic leukemia (CMML), or exposure to potentially leukemogenic agents). The risk categories of the patients were based on the European LeukemiaNet 2022 (ELN 2022) risk category Guidelines for AML (25). The study design is shown in **Figure 1A**.

This study was approved by the Institutional Review Board (approval code: 2019ZDSYLL211-P01) and all patients provided written informed consent before enrollment according to institutional policies and the declaration of Helsinki.

### Procedures

Induction therapy in a 28-day cycle consisted of Aza (75mg/m^2^/d on days 1-5 subcutaneous) administered in combination with HIA regimen [HHT 2mg/m^2^/d on days 4-8 intravenous over 3 hours, idarubicin 6mg/m^2^/d on days 4-6 intravenous, and cytarabine 100mg/m^2^/d on days 4-10 intravenous] for patients age<60 or age≥60 eligible for IC (**Figure 1A**). Patients who did not achieve a CR or CR with incomplete blood count recovery (CRi) following the first cycle could receive a second cycle at the same doses. The patients who did not reach the CR/CRi after the second cycle were withdrawn from the study. Post-remission therapy for patients who received CR/CRi, high-dose cytarabine was given as consolidation therapy followed by hematopoietic stem cell transplantation for those candidates (**Figure 1B**).

In this study, to prevent infection of patients with granulocytopenia, quinolones antibiotics, voriconazole (or echinocandins) were considered during the induction until the recovery of neutrophils, and laminar flow air cleaner beds were utilized to reduce opportunistic infection.

Response to therapy was monitored by analysis of blood and bone marrow (BM) aspirates. Response assessment was done at the end of cycles 1 and 2, after every 2 cycles of consolidation and every 3 months during maintenance to confirm ongoing response. Responses were categorized based on the revised International Working Group criteria for AML (26).

Minimal residual disease (MRD) assessment by multicolor flow cytometry (MFC) was done on pretreatment BM and all subsequent BM examinations as previously described (27). BM samples were obtained at diagnosis and evaluated for the presence of cytogenetic and molecular aberrations. Real-time quantitative PCR (RT-qPCR) was used to detect the gene rearrangements. For documentation of mutations, the entire coding sequences of 58 leukemia driver genes (**Table S1**) known to be frequently mutated in myeloid malignancies were sequenced with targeted leukemia exome-seq panel.

Adverse events (AEs) and laboratory values, graded according to the Common Terminology Criteria for AEs version 4.0, were evaluated at least once every cycle during induction and consolidation and then at least every 3 months during the maintenance.

### Sample size estimation

In this trial, we applied minimax Simon’s two-stage design method (28) to determine the sample size. A composite CR (CR or CRi) rate ≤ 65.0% (p0) would be unacceptable (null hypothesis). While a composite CR rate of higher than 90.0% (p1) would support the ongoing trial for further exploration. Meanwhile, we used an estimation power of 80.0%, with a significance level of 5.00% to test the hypothesis.

15 participants were enrolled in the 1st stage, with additional 3 participants in the 2nd stage. This trial would be discontinued if 12 or fewer achieved composite CR in the 1^st^ stage. The enrollment procedure of the 2nd stage would be held until the interim analysis result of the 1st stage. If this trial continued, the activity of the combination therapy would be considered a null hypothesis if less than 14 out of 18 enrolled cases achieved composite CR. Finally, considering a 10% dropout rate, the estimated sample size was 20 AML patients.

### Clinical endpoint and assessments

The primary endpoint was the composite CR (CR/CRi). Criteria of CR were as follows: (1) no signs of leukemia after treatment, (2) fewer than 5% blasts in the BM, (3) absolute neutrophil count (ANC) >1000/μL and platelet count > 100,000/μL, (4) absence of the extramedullary tumor. CRi was defined as fulfilling all CR criteria except for ANC <1000/μL or platelet count <100,000/μL in peripheral blood. Secondary endpoints were overall survival (OS) defined as the time from study entry to death from any cause, relapse-free survival (RFS) defined as the time from achieving a CR until disease recurrence or death, AEs (including hematological and non-hematological AEs defined as any unfavorable and unintended signs including an abnormal laboratory finding, symptom, or disease). Treatment failure was defined as not achieving CR or CRi after two cycles of induction therapy.

Prespecified correlative assessments included targeted gene panel sequencing to assess associations between somatic mutation patterns and therapeutic responses as well as disease progression.

### Inclusion and exclusion criteria

Eligible patients were aged 18-70 years and ND, previously untreated *de novo* AML according to the criteria of WHO (24). The including criteria were as follows: (1) Eastern Cooperative Oncology Group performance status (ECOG) ≤2 (5-point scale), (2) Normal left ventricular ejection fraction ≥50% (echocardiography), (3) Adequate hepatic function (alanine aminotransferase and aspartate aminotransferase concentration < 2 times of upper limit of normal, serum bilirubin concentration ≤35 μmol/L, (4) Adequate renal function (serum creatinine ≤150 μmol/L), (5) Able to understand and provide the written consent. Exclusion criteria were as follows: (1) Patients diagnosed with acute promyelocytic leukemia (M3), (2) Patients with a history of the antecedent myeloid disease, including MDS, MPN, or CMML, (3) Patients were positive for HIV (human immunodeficiency virus), (4) A history of chronic stable angina or congestive heart failure with an ejection fraction < 50%, or COPD (chronic obstructive pulmonary disease) with FEV (forced expiratory volume) less than 65% in 1 second, (5) Uncontrollable infection (bacterial, fungal or viral) or respiratory disease (requiring continuous supplementary oxygen), (6) Mentally unhealthy or unable to sign the informed consent, (7) lactating and pregnant women (women of childbearing age would receive the test of β-subunit of human chorionic gonadotropin), (8) Other medical or psychological diseases which investigators believed were not suitable to enter the trial.

### Targeted leukemia exome-seq panel for gene mutation screening in AML patients

A leukemia targeted-exome-seq panel including 58 leukemia driver genes was used for screening the gene mutations in 20 enrolled patients by next-generation sequencing (NGS) before and after the induction therapy (29, 30). The target genes in the panel are listed in **Table S1**.

Agilent SureSelect Human All Exon V4+UTRs (Agilent) was used for the coding exons plus UTRs of target genes. Probes for each exon of each target gene are designed by the National Center for Biotechnology Information (https://www.ncbi.nlm.nih.gov/). The targeted exome-seq method is performed as reported (31). Briefly, the genomic DNA was isolated from BM samples with the genomic DNA isolation kit (Qiagen, Hilden, Germany). All DNA samples were sheared with a Covaris E220 instrument generating approximately 260 bp DNA fragments. The fragmented DNA was processed into Illumina-compatible sequencing libraries using Kapa Hyper Prep Kit (Illumina, San Diego, CA, USA). Each library was uniquely barcoded and captured by the leukemia panel probes, followed by PCR amplification and sequencing on a HiSeq 2500 (Illumina) with 2x100 bp reads. The sequencing reads were aligned to the human genome by following Broad Institute’s GATK best-practice pipeline to call germline short variants (SNPs and Indels). Called variants were annotated using ANNOVAR (version 2.3). Exonic variants with exonic, nonsynonymous, stop-gain, or stop-loss, novel SNPs, and with predicted deleterious/damaging functions were manually surveyed by the Integrative Genomics Viewer (IGV) to confirm.

The association of gene mutations with clinical response, relapse, and risk status was analyzed with R 4.0 software and depicted as a waterfall figure. The association of the gene mutations with OS and RFS was also evaluated by the Kaplan-Meier method.

### Minimal residual disease detection

MRD assessment by MFC was done on pretreatment BM and all subsequent BM examinations with an assay sensitivity of 0.01% (32). A wide antibody panel of molecular antigens (Beckman, USA) used for MRD detection of AML were as follows: CD34, CD117, CD13, CD33, CD7, CD56, CD10, CD19, CD38, HLA-DR, and CD45. After incubating the BM samples with monoclonal antibodies for 15 minutes, erythrocytes were lysed using lysing solution (QIAGEN Science, USA). Afterward, to remove excess antibodies and lysed red blood cells, the samples were washed twice with 2 mL PBS (phosphate-buffered saline) buffer (pH 7.4) and centrifuged (600g×5 minutes, room temperature). The samples were then measured using the NAVIOS system (Beckman Coulter) and analyzed for at least 50, 000 events. All samples were analyzed on the Kaluza analyzing platform (Beckman, USA). The MRD detected by MFC was defined as at least 20 clustered cells showing myeloid scattering properties, leukemia-associated immunophenotype (LAIP) characteristics, and a minimum of 1×10^6^ white blood cells (WBC) were required for MRD monitoring to achieve theoretically maximum sensitivity of 2×10^-5^. After checking cell viability and debris through FSC/SSC scatter plot, the main gating strategy for detecting MRD was as follows: 1. CD45/SSC plot was used to show the whole WBC, 2. CD34+, CD117+, or CD133+ fractions were used to show progenitor/primitive cells, 3. CD45dim/SSC low plot was used to detect immature cells (or CD45high/CD34 negative plot if cells are more mature), 4. within the above fractions, the percentage of aberrancies was defined by the plot of myeloid markers (CD33 and CD13) in combination with lineage markers (CD7, CD56). The negative status of MRD was defined as MRD less than 0.1%, while the positive status of MRD was defined as 0.1% or higher. Two investigators were joined to evaluate all the samples to avoid bias.

### Fusion gene panel screening in AML patients

A leukemia fusion gene panel including 47 fusion genes was used for screening all 20 enrolled patients at the time of diagnosis. The fusion genes in the panel are listed in **Table S2**.

BM samples were obtained at diagnosis and evaluated for the presence of gene rearrangement. The isolation of total RNA, cDNA synthesis, and RT-qPCR was used to detect the gene rearrangements, according to the manufacturer’s (Zeesan Biotech, China) protocol. The RT-qPCR was performed on the ABI7500 platform (thermos scientific, USA) with specific primers for the fusion genes. Each procedure of PCR included a standard curve (a serial dilution of plasmids from 10^6^ to 10^2^), negative controls, and patients’ samples. Negative controls and patients’ samples were in duplicate and standard curves in triplicate. The sensitivity of RT-qPCR was 10^-5^ at the cellular level. Patients with specific positive fusion genes were further monitored after the induction therapy by the RT-qPCR method, and the absolute copy number of fusion gene transcripts was normalized to *ABL1*, expressed per 10^5^ copies of *ABL1*.

### Statistical analysis

Lower and upper limits of the 95% confidence interval for a proportion were calculated by the Wilson method (continuity correction). Survival was estimated by the Kaplan-Meier method, which was done by STATA 14.0 and GraphPad 8.0 software. A two-sided α level of 0.05 was considered statistically significant.

## Results

### Baseline characteristics of the enrolled patients

Between Jan 2020 and Sep 2022, 26 patients were screened for eligibility, and 20 previously untreated *de novo* AML patients were enrolled (9 were male, 11 were female). The median age was 50 years (range 18-70) (3 of 20 patients ≥60 years, 17 of 20 patients <60 years). The enrolled patients were classified into favorable-risk (9/20), intermediate-risk (8/20), and poor-risk(3/20)according to the ELN 2022 risk category Guidelines for AML. The most frequently mutated genes were *FLT3* (ITD/TKD) (20.0%, 4/20), *DNMT3A* (20.0%, 4/20), *NRAS* (15.0%, 3/20), and *KRAS* (15.0%, 3/20). The baseline characteristics and demographic of the enrolled patients are presented in **Table 1**.

**Table 1:** Clinical features, classification, and treatment responses of the enrolled patients.

ID	Sex	Age	Risk category	FAB type	Response	The frequency of gene variants detected with next-generation sequence panel			
						Mutation(nucleotide)	Mutation(amino acid)	Before induction(VAF, %)	After induction(VAF, %)
1	F	70	Intermediate	M1	CRi	WT1: c.1140dup	p. Ser381ValfsTer4	34.40	3.81
						WT1: c.1138C>G	p.R380G	34.65	3.82
2	M	59	Intermediate	M5	CR	No gene mutation was found			
3	F	34	Poor	M5	NR	SETBP1: c.2602G>T	p.D868Y	45.77	NA
						U2AF1: c.101C>T	p.S34F	45.61	NA
4	M	60	Favorable	M2	CRi	NRAS: c.35G>A	p.G12D	2.16	0.00
						NRAS: c.183A>C	p.Q61H	2.09	0.00
5	M	54	Intermediate	M5	CR	KRAS: c.38G>A	p.G13D	31.88	0.00
6	F	22	Favorable	M4	CR	FLT3-ITD		22.19	0.00
						KRAS: c.35G>A	p.G12D	4.61	0.00
						NRAS: c.182A>G	p.Q61R	4.56	0.00
7	M	41	Favorable	M4	CR	CSF3R: c.2346dup	p.Ser783GlnfsTer6	45.01	0.00
						WT1: c.1385G>A	p.R462Q	41.09	0.00
8	M	57	Poor	M5	CR	TP53: c.527G>A	p.C176Y	67.54	0.00
9	M	48	Intermediate	M2	CR	DNMT3A: c.2645G>A	p.Arg882His	83.85	0.00
10	F	53	Intermediate	M4	CR	FLT3: c.2516A>G	p.D839G	21.78	0.00
						FLT3: c.2522A>C	p.N841T	2.10	0.00
						FLT3: c.2508_2510del	p.Ile836del	21.58	0.00
11	F	43	Favorable	M4	CR	FLT3: c.2523C>A	p.N841K	12.64	0.00
						FLT3: c.2516A>C	p.D839A	2.00	0.00
						FLT3: c.2508_2510del	p.Ile836del	6.09	0.00
						KIT: c.2466T>G	p.N822K	15.51	0.00
12	F	21	Favorable	M2	CR	CEBPA: c.937_939dup	p.Lys313dup	34.29	Positive*
						GATA2: c.952G>A	p.A318T	35.95	Positive*
13	F	18	Intermediate	M5	CRi	No gene mutation was found			
14	M	49	Favorable	M2	CRi	KIT: c.2447A>T	p.D816V	42.67	0.00
15	F	47	Favorable	M4	CR	NRAS: c.35G>A	p.Gly12Asp	7.40	0.00
						PTPN11: c.1520c>A	p.Thr507Lys	2.00	0.00
						NPM1: c.2446C>T	p.Arg816Ter	30.80	0.00
						DNMT3A: c.2645G>A	p.Arg882His	42.20	0.00
16	M	35	Poor	M2	CR	U2AF1: c.101C>T	p.S34F	38.90	29.17
						BCOR: c.2488_2489delCT	p.Ser830CysfsTer6	38.95	8.67
17	F	53	Intermediate	M1	CR	DNMT3A: c.2645G>A	p.R882H	24.19	30.18
						IDH2: c.419 G>A	p.R140Q	11.65	0
18	M	58	Favorable	M2	CR	EZH2: c.794A>G	p.D265G	46.67	0
						EZH2: c.236_237insCG	p.Thr80G1yfsTer7	41.98	0
						GATA2: c.821_822insCCCCTGGGGGGACC	p.Ala275ProfsTer56	45.04	0
						KRAS: c.38G>A	p.G13D	16.75	0
19	F	62	Favorable	M5	CRi	TET2: c.4006delA ()	p.Thr1336HisfsTer27	48.53	2.74
						TET2: c.3579T>G	p.C1193W	48.37	7.10
						TET2: c.4892_4893delAT	p.Tyr1631SerfsTer29	0	2.85
						NPM1: c.860_863dupTCTG	p.Tp288CysfsTer12	47.55	2.13
						PTPN11: c.226G>A	p.E76K	37.75	0
						DNMT3A: c.2645G>A	p.R882H	0	18.44
						ASXL1: c.2756_2757delAT	p.lle919ThrfsTer4	13.76	0
20	F	51	Intermediate	M5	CRi	TP53: c.818G>A	p.R273H	8.61	0
						FLT3: c.2503G>C	p.D835H	8.54	0
						FLT3: c.2516A>G	p.D839G	7.79	0

F, female; M, male.

FAB classification=French–American–British classification.

The risk category was evaluated based on the classification of European Leukemia Net 2022.

VAF, variant allele fraction.

NA, Not available.

CR, complete remission.

CRi, complete remission with incomplete blood count recovery.

*Detected by the Sanger sequencing method.

### Clinical efficiency

**Figure 2:**
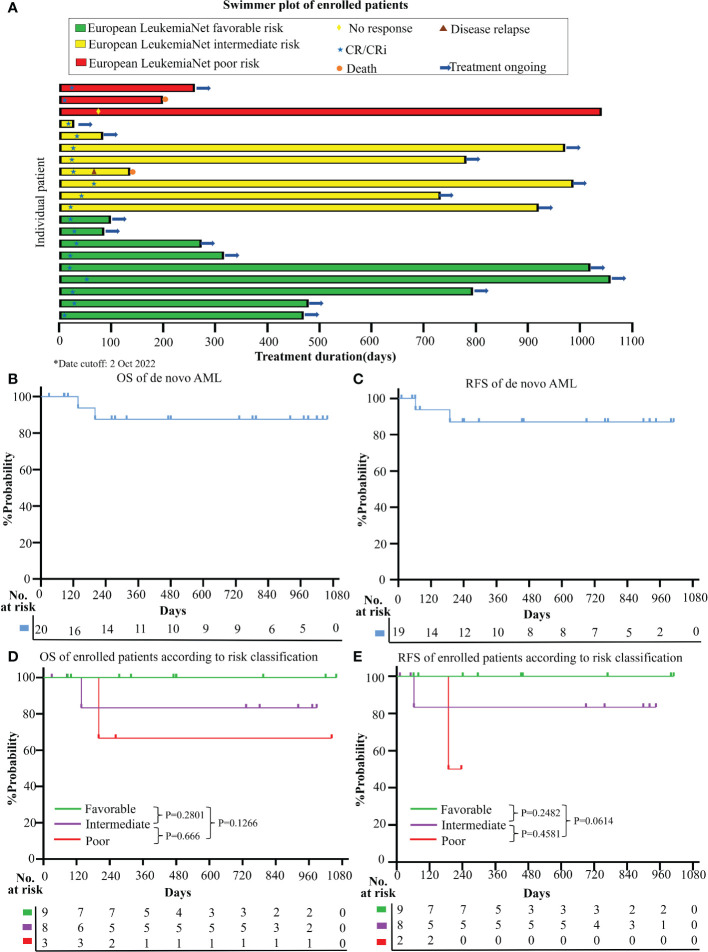
Clinical responses and survival **(A)** Treatment durations, hematological responses, and patient dispositions of this clinical trial (date cutoff at 2 Oct 2022); Kaplan-Meier method was used to estimate the overall survival (OS) **(B)** and relapse-free survival (RFS) **(C)** for enrolled AML patients; OS curve **(D)** and RFS curve **(E)** according to different risk categories (Favorable+ Intermediate versus. Poor risk group; risk classification was based on ELN 2022 category).

CR/CRi was achieved in 95.0% (19/20; [95%CI, 73.1% to 99.7%]) of the enrolled patients (**Figure 2A**). 94.7% (18/19) reached CR/CRi after the 1st cycle of the induction therapy and the median time to the first CR/CRi was 25.0d (IQR, 21.0 to 33.0). At the date cutoff (Oct 2, 2022), the median follow-up was 733 days (range, 30 to 1059) for all enrolled patients, and no patient was lost to the follow-up. The median OS and RFS were not reached and the estimated 2-year OS and RFS were 87.5% (95%CI, 58.6% to 96.7%) and 87.1% (95%CI, 57.3% to 96.6%), respectively (**Figures 2B, C**).

Based on ELN2022 risk classification, CR/CRi rate was achieved in 100% (17/17) of the favorable and intermediate risk-group, and 66.7% (2/3) in the poor risk-group. No CR/CRi rate difference was observed between the favorable and intermediate versus poor-risk groups (P=0.15). Besides, no survival difference was observed among the different risk groups in both OS (favorable vs intermediate, P=0.2801; intermediate vs poor, P=0.666; favorable vs poor, P=0.1266) and RFS (favorable vs intermediate, P=0.2482; intermediate vs poor, P=0.4581; favorable vs poor, P=0.0614) (**Figure 2D, E**).

### Efficacy of the novel regimen in the patients with genetic defects in leukemia driver genes

**Figure 3:**
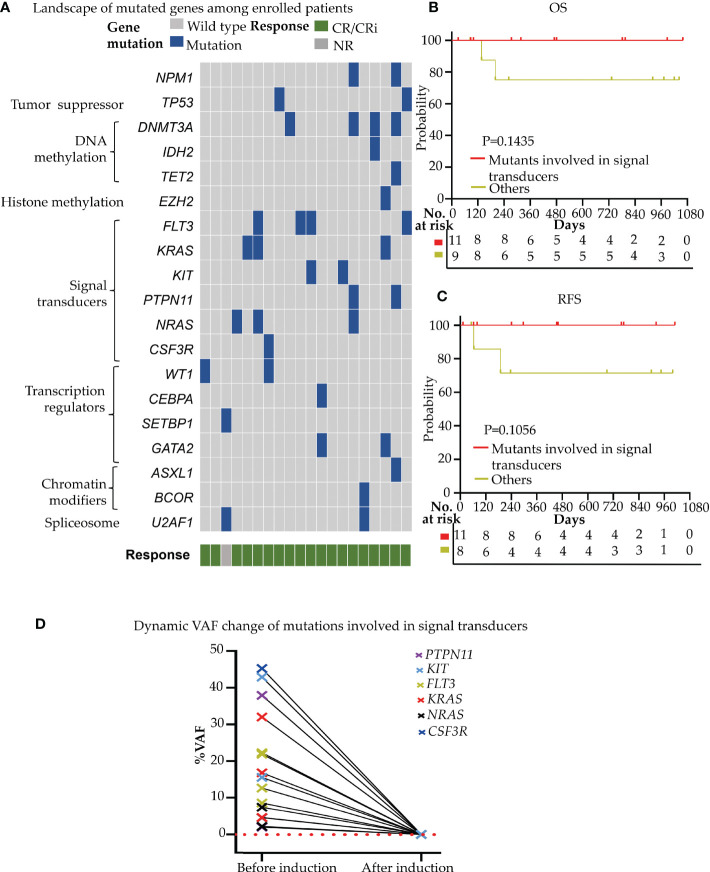
Gene mutation profiling and effect on the clinical efficacy of Aza+HIA regimen **(A)** Landscape of mutations detected in 18 of 20 enrolled patients. Each row represents a gene, and each column corresponds to a participant. The sidebar plots the number of mutations detected for each gene; **(B, C)** comparison of the OS **(B)** and RFS **(C)** in patients harboring mutations involved in signal transducers versus those without the mutants in the trial; **(D)** VAF clearance of mutated genes involved in signal transducers (*FLT3*, *KRAS*, *NRAS*, *KIT*, *PTPN11*, *CSF3R*) among 11 available patients (before induction versus. after reached CR/CRi).

We detected 36 mutants in 19 of 58 leukemia driver genes in all enrolled patients at the timepoint of enrollment. The median mutation number/patient was 2.0 (range, 0-5), and 18 of 20 (90.0%) patients had more than one mutation (≥1) (**Figure 3A**). More than half of the patients (11/20) harbored the mutated genes involved in signal transducers (*FLT3*, *KRAS*, *NRAS*, *KIT*, *PTPN11*, *CSF3R*; 11/20, 55.0%), and all these patients achieved CR/CRi (11/11, 100%) upon the novel combined therapy (**Figure 3A**). Also, the 11 patients who harbored mutations involved in the signal transducers acquired an improved trend in both OS (HR=0.126, 95%CI, 0.00786 to 2.021; P=0.1435) (**Figure 3B**) and RFS (HR=0.0993, 95%CI, 0.00606 to 1.629; P=0.1056) (**Figure 3C**), although no significance was observed compared to that without the mutants. It is worth noticing that a high mutant’s clearance with VAF (variant allele frequency) was dramatically reduced (to undetectable) (**Figure 3D**) after the induction therapy among all of the 11 cases. These data indicate that the patients with the gene mutation of the signal transducers had a rapid and high clinical response to the Aza+HIA regimen.

We also screened the 47 common leukemia fusion genes in the trial and observed the efficacy in the patients with leukemia fusion genes. We identified 3 patients harboring the *MLL-AF9* fusion gene and 3 patients having *CBF-β -MYH11* fusion gene. All 3 patients with the *MLL-AF9* fusion gene reached complete molecular remission (<0.1%, undetectable) after 1 cycle of induction. 2 of 3 patients with the *CBF-β -MYH11* fusion gene reached complete molecular remission (<0.1%, undetectable), and the expression level of the *CBF-β -MYH11* fusion gene in 1 patient decreased over 1000-fold at the molecular level (512.16% to 0.28%) after 1 cycle of induction. These results indicated that the patients with the fusion genes could also achieve clinical and/or molecular remission upon the Aza+HIA induction treatment.

### Efficacy of the novel combination regimen for MRD

**Figure 4:**
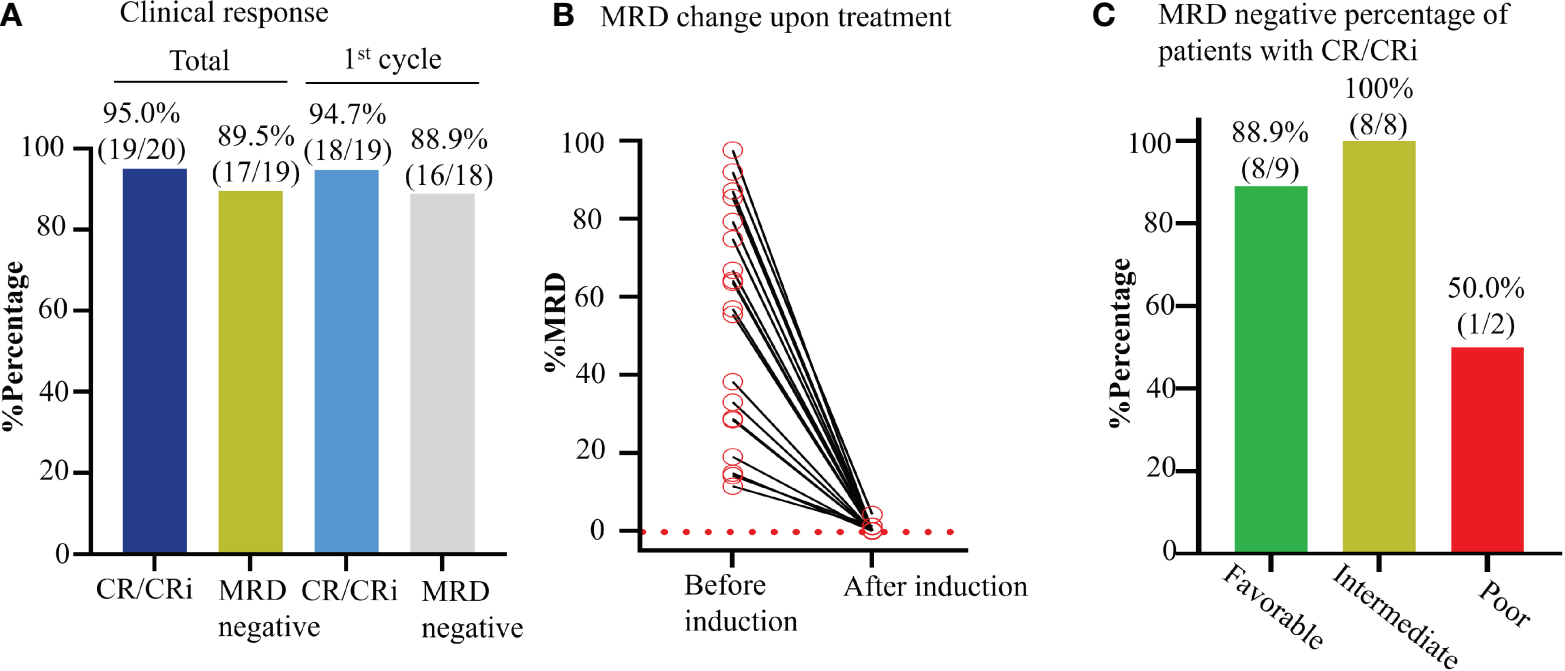
Effect of Aza+HIA on the MRD in the clinical trial. **(A)** Bar plot of CR rate of enrolled AML patients and MRD negative percentage of patients with CR/CRi, in total and after 1^st^ cycle of Aza+HIA treatment. **(B)** Clearance of MRD upon the treatment in 19 available CR/CRi patients. **(C)** Effect of the regimen on the MRD in the patients (CR/CRi) with different risk statuses.

The MRD negative was 89.5% (17/19; 95% CI, 65.5% to 98.2%) in patients who reached CR/CRi, and 88.9% (16/18; 95% CI, 63.9% to 98.1%) in patients who reached CR/CRi after the 1^st^ cycle of induction therapy (**Figures 4A, B**). Also, MRD was negative in 88.9% of patients with favorable-risk (8/9; 95%CI, 50.7% to 99.4%), 100% (8/8; 95%CI, 59.8% to 99.9%) with intermediate-risk, and 50.0% (1/2; 95%CI, 2.67% to 97.3%) with poor-risk (**Figure 4C**). Moreover, MRD negative was achieved in one patient after one cycle of consolidation therapy. These data indicated the novel regimen could efficiently eradicate the MRD to reach the deep and rapid molecular response in *de novo* AML.

### Safety

Common non-hematological AEs are summarized in **Table 2**, infection (grade 1-4) was the most common non-hematological AE, presenting 75.0% (15/20) in the whole cohort, and infection of grade 3 or higher occurred in 60.0% of patients (12/20). Other AEs of grade 3 or higher that occurred in the whole cohort included fatigue (1/20, 5.0%), decreased appetite (1/20, 5.0%), and hepatic dysfunction (1/20, 5.0%). No grade 5 AEs or tumor lysis event was observed, and no cardiac AE was observed.

**Table 2:** AEs of Aza+HIA regimen in enrolled patients.

		All enrolled patients (n=20)	
Non-Hematologic AEs		All grades	Grades ≥3
	Constipation	5 (25.0)	0
	Diarrhea	2 (10.0)	0
	Vomiting	7 (35.0)	0
	Hypokalemia	5 (25.0)	0
	Peripheral edema	0	0
	Fatigue	10 (50.0)	1 (5.0)
	Decreased appetite	10 (50.0)	1 (5.0)
	Hemorrhage	0	0
	Cardiac arrhythmia	0	0
	Infection	15 (75.0)	12 (60.0)
	Lung infection	12 (60.0)	9 (45.0)
	Gastrointestinal infection	1 (5.0)	1 (5.0)
	Perirectal abscess	1 (5.0)	1 (5.0)
	Skin infection	1 (5.0)	1 (5.0)
	Sepsis	2 (10.0)	2 (10.0)
	Nausea	9 (45.0)	0
	Hepatic dysfunction	3 (15.0)	1 (5.0)
	Fever	14 (70.0)	0
Hematologic AEs	While blood cell count decrease	20 (100)	20 (100)
	Neutrophil count decrease	20 (100)	20 (100)
	Platelet count decrease	20 (100)	20 (100)
Early mortality	Died within 4 weeks	0	0
	Died within 8 weeks	0	0

Data are n (%), unless otherwise stated.

As for the hematological toxicity, the most common grade 3-4 hematological AEs were neutropenia (100%, 20/20) and thrombocytopenia (100%, 20/20). Among 19 patients who reached CR/CRi, the median duration of neutropenia (ANC ≤500/μL) was 12d (IQR, 11 to 15), and the median duration of thrombocytopenia (absolute platelet count ≤50000/μL) was 14d (IQR, 13 to 16).

It is noticed that no patients discontinued the induction treatment or required dose reduction due to hematological or non-hematological AEs. In addition, no patient died within 8 weeks of the treatment.

## Discussion

In the past three decades, standard induction regimens used for patients younger than 60 years or fit older patients eligible for IC are based on a backbone of cytarabine plus an anthracycline (idarubicin/daunorubicin/mitoxantrone). The current NCCN (National Comprehensive Cancer Network) Guidelines for AML recommend that idarubicin 12 mg/m^2^ or daunorubicin 60-90 mg/m^2^ daily for 3 days plus cytarabine 100-200 mg/m^2^ daily for 7 days (so-called “3+7” regimen). The CR rates for patients younger than 50 years have consistently been in the range of 60-80% in most large cooperative group trials with a “3+7” regimen (33).

In recent years, many clinical studies tried to find novel regimens with higher efficacy than the standard 3 + 7 regimen. The CR/CRi rate after induction therapy adding midostaurin (a multi-kinase inhibitor) to IC is 75.8% in patients aged <60 years with FLT3-ITD positive AML (34). It was reported that a CR rate of 82% and estimated 5-year survival of 52% in patients with ND AML (aged <65 years) treated with FLAG-IDA and GO (35). The addition of cladribine was associated with higher rates of CR (67.5% vs 56% [*P* = 0.01]) and 3-year survival (45% vs 33% [*P* = 0.02]) compared with “3+7” regimen alone (36) and also improved outcome in *FLT3*-mutated AML (37). A study on IDH1/2 inhibitors combined with IC in patients with ND AML showed that CR/CRi rates were 72% and 63%, respectively. 16/41 (39%) receiving ivosidenib had *IDH1* mutation clearance and 15/64 (23%) receiving enasidenib had *IDH2* mutation clearance; 16/20 (80%) and 10/16 (63%), respectively, became negative for MRD by MFC (38).

As a hypomethylating agent, Aza has emerged as a backbone for novel combinations in treating AML. Recent years have observed various “Aza+ X” combination schemes and many of them reported promising clinical responses (Aza+ lenalidomide (7), Aza+ histone deacetylase inhibitor (8), Aza+ venetoclax (9), Aza+ FLT3 inhibitor (10), Aza+ anti-CD33 antibody (11), etc.). As an anti-apoptotic protein, MCL1 plays a key role in promoting the cell survival of AML cells (39). Both Aza and HHT were reported to downregulate the MCL1 (40, 41). Therefore, we designed “Aza plus HHT” combination-based regimen and evaluated the clinical efficiency of the scheme in AML.

Our recently published clinical trial demonstrated the promising therapeutic responses in elderly AML patients or ineligible for IC treated with Aza combined with HAG (HHT, low-dose cytarabine, G-CSF) (23). To our knowledge, this is the first clinical trial to evaluate the efficacy and safety of the combination of Aza and HIA regimen in young and/or older patients fit for IC. This study showed a high CR/CRi rate of 95%, and 94.7% reached CR/CRi after the 1st cycle of induction therapy (median time to CR/CRi was 25.0d), indicating a promising clinical response compared with the previous reports (22). Moreover, we observed that 89.5% of patients were MRD negative and 88.9% reached MRD negative after 1st cycle of treatment, indicating the deep remission and rapid clearance of MRD upon the therapy of the Aza+HIA regimen.

A previous study has reported that HHT-based regimens, including HDA (HHT, daunorubicin, cytarabine) and HAA (HHT, aclarubicin, cytarabine), benefit AML patients more rather than DA (daunorubicin, cytarabine, “3+7”) regimen without increased toxicity (22). Our clinical trial expanded the observation and found that adding Aza into the HIA regimen dramatically increased the clinical efficacy in AML patients.

In this trial, the Aza+HIA regimen was well tolerated, and no patients died within 8 weeks of the induction treatment. The most common grade 3 or higher non-hematological AEs was infection (60.0%), but no case discontinued the treatment. These data indicated that the Aza+HIA regimen had lower cardiac toxicity and hematological suppression than daunorubicin in the standard regimen.

Our results demonstrated that the Aza+HIA regimen yielded a much higher CR/CRi rate (95%) than the CR rates of 73%, 67%, and 61% for HAA, HDA, and DA induction regimens in a published study on patients aged <60 with *de novo* AML (22), indicating that adding Aza to HHT-based regimen significantly increases the clinical responses and outcome for *de novo* AML patients. Additionally, the estimated 2-year OS and 2-year RFS were 87.5% and 87.1%, respectively. This study indicated that medically fit (eligible for receiving IC) *de novo* AML patients can be treated through the Aza+HIA regimen, strive to reach CR/CRi through an induction course, and eliminate leukemia cells to the greatest extent (deep remission of disease), to shorten the number of chemotherapy cycles and improve the quality of life for AML patients.

Our study also indicated that the dosage of idarubicin could be reduced (6mg/m^2^/d of idarubicin in this trial) when in combination with Aza and HHT. The dose reduction of anthracene drugs could lead to less cardiotoxicity (42). Correspondingly, no cardiac arrhythmia event (grade1-4) was observed in this trial, highlighting the potential treatment usage in clinical practice. This study had potential limitations. The data were collected in a single center, and the sample size was relatively small. However, the present results might provide preliminary information for further design of a phase 3 trial.

## Conclusions

In summary, this trial demonstrated that the Aza+HIA regimen is an effective first-line therapy with high efficacy and well-tolerance for previously untreated young or fit older patients with AML. This study provides the support for further larger samples in a randomized controlled trial.

## Data availability statement

The original contributions presented in the study are included in the article/**Supplementary Materials**. Further inquiries can be directed to the corresponding author.

## Ethics statement

The studies involving human participants were reviewed and approved by Institutional Review Board of Zhongda Hospital, Affiliated to Southeast University. The patients/participants provided their written informed consent to participate in this study.

## Author contributions

ZG designed the clinical trial, guided and organized the performance of the clinical trial, guided the targeted leukemia exome-seq panel, instructed the laboratory experiments, and edited the manuscript. JL performed the clinical trials, collected and analyzed the clinical data, wrote, and edited the manuscript. YQH: Performed the clinical trials, collected and analyzed the clinical data, and data curation. YH: Performed the clinical trials, and collected and analyzed the clinical data. YG performed the clinical trials and data curation. CS participated in the guidance of the data analysis and edited the manuscript. All authors contributed to the article and approved the submitted version.
