# Supplementary material for: High efficacy of azacitidine combined with homoharringtonine, idarubicin, and cytarabine in newly diagnosed patients with AML: A single arm, phase 2 trial
Source: Front Oncol. 2022 Dec 8;12:1069246. doi: 10.3389/fonc.2022.1069246 (PMC9773133; doi:10.3389/fonc.2022.1069246)
Supplement: Supplementary file 1 [file DataSheet_1.pdf]

Supplementary Material

Supplementary Tables

Table S1 The Leukemia Panel for next-generation sequencing

ABL1	BRAF	CEBPA	ETV6	HRAS
ANKRD26	CALR	CSF3R	EZH2	IDH1
ASXL1	CBL	CUX1	FLT3	IDH2
ATRX	CBLB	DDX41	GATA1	IKZF1
BCOR	CBLC	DNMT3A	GATA2	JAK2
BCORL1	CDKN2A	ETNK1	GNAS	JAK3
KDM6A	NPM1	PTEN	SMC1A	TP53
KIT	NRAS	PTPN11	SMC3	U2AF1
KMT2A	PDGFRA	RAD21	SRSF2	WT1
KRAS	PHF6	RUNX1	STAG1	ZRSR2
MPL	PIGA	SETBP1	STAG2	
NF1	PPM1D	SF3B1	TET2	

Table S2 The Leukemia Panel for fusion gene screening

TEL-ABL1	AML1-MDS1	PLZF-RARα	MLL-AF17
TEL-JAK2	E2A-HLF	NUP98-HOXA9	NUP98-HOXA11
FIP1L1-PDGFRA	DEK-CAN	NUP98-HOXA13	NUP98-HOXC11
ETV6-PDGFRA	NPM-RARα	NUP98-HOXD13	NUP98-PMX1
STRN-PDGFRA	PML-RARα	NUP98-TOP1	NPM-MLF1
KIF5B-PDGFRA	MLL-AF10	TEL-PDGFRB	MLL-AFX
SIL-TAL1	MLL-AF1p	RNP1-EV11	CBFB-MYH11
MLL-AF6	MLL-AF9	AML1-CLCA2	E2A-PBX1
MLL-ELL	MLL-AF1q	NPM-ALK	AML1-ETO
MLL-AF4	MLL-ENL	TEL-AML1	BCOR-RARα
BCR-ABL	TLS-ERG	STAT5B-RARα	FIP1L1-RARα
AML1-MTG16	SET-CAN	NUMA1-RARα	
